# A taxonomy of visualization tasks for the analysis of biological pathway data

**DOI:** 10.1186/s12859-016-1443-5

**Published:** 2017-02-15

**Authors:** Paul Murray, Fintan McGee, Angus G. Forbes

**Affiliations:** 1 Electronic Visualization Laboratory, University of Illinois at Chicago, Chicago, IL USA; 2 eScience Group, Environmental Research and Innovation (ERIN) department, Luxembourg Institute of Science and Technology, Esch/Alzette, Luxembourg

**Keywords:** Biological pathways, Pathway visualization, Task taxonomy

## Abstract

**Background:**

Understanding complicated networks of interactions and chemical components is essential to solving contemporary problems in modern biology, especially in domains such as cancer and systems research. In these domains, biological pathway data is used to represent chains of interactions that occur within a given biological process. Visual representations can help researchers understand, interact with, and reason about these complex pathways in a number of ways. At the same time, these datasets offer unique challenges for visualization, due to their complexity and heterogeneity.

**Results:**

Here, we present taxonomy of tasks that are regularly performed by researchers who work with biological pathway data. The generation of these tasks was done in conjunction with interviews with several domain experts in biology. These tasks require further classification than is provided by existing taxonomies. We also examine existing visualization techniques that support each task, and we discuss gaps in the existing visualization space revealed by our taxonomy.

**Conclusions:**

Our taxonomy is designed to support the development and design of future biological pathway visualization applications. We conclude by suggesting future research directions based on our taxonomy and motivated by the comments received by our domain experts.

## Background

Understanding complicated networks of biomolecular entities and interactions is essential to solving contemporary problems in modern biology, especially in computational domains such as systems biology [1]. Networks of biomolecular interactions are represented as graph models referred to as *pathways*. Pathways are curated subsets of a theoretical graph of all known biomolecular entities and events that occur on the cellular level, and a given pathway usually represents a particular biological process, such as mitosis, that is relevant within a given research context.

Pathways are modeled as labeled graphs of entities, relationships, and meta-data. An entity is a component of a pathway such as a gene, a gene product (such as a protein), a complex of proteins, a small biomolecule, or even another pathway. Edges between vertices in this graph can be directed or undirected, can involve multiple entities in one relationship, and can represent a wide range of biological relationships. Meta-data can include external information such as experimental data, as well as the *provenance* of the information related to a particular entity or relationship. Provenance is typically a list of records, such as publications, that reflects the collective history of research related to a given entity or relationship. Provenance is essential to the field of bioinformatics, as the “ground truth” related to any given entity is not immutable, and can be derived from a potentially large and evolving history of research.

Researchers who work with pathway data are confronted with a number of challenges. Pathway files may contain hundreds or thousands of entities that are connected by a wide variety of relationship types. For instance, the *BioPax* [2] specification contains a “Transport” class, which is one of four types of “Conversion,” which in turn is one of five different types of “Interaction,” which, finally, is one of four types of “Entity.” The *BioPax* schema is itself a reflection of the complexity of information that can exist within bio-chemical pathway datasets.

Participants in a pathway — genes, proteins, and other molecules within a cell — can act as inputs or outputs to multiple interactions, and the set of relationships between biochemical interactions inherently includes feedback loops and other complex relationships. Importantly, reactions and other interactions can have a “cascading” effect, where one interaction will inhibit or promote the effect of another. Molecular activation pathways also have an inherently dynamic quality, which can limit the utility of static (i.e., non-interactive) graph representations [3]. Understanding these complex and dynamic relationships while also enabling researchers to see higher order patterns is a significant challenge to modern bioinformatics research [4].

Pathway diagrams are used in two contexts: for the presentation of results, and as an active (and interactive) part of the process of data analysis. In the presentational sense, pathway diagrams can contextualize a set of biological processes within a cell, and in these contexts will often show the location of cellular membranes and other large cellular structures to help to provide a frame of reference for the viewer. Ideally, a pathway diagram — when used in a presentational context — allows a viewer to efficiently understand a complex set of biological relationships. While pathway diagrams may be useful for presenting and contextualizing a set of results in a research or educational context, they are also an important part of *in situ* analyses.

For example, metabolic activation networks are of critical importance to cancer researchers, who hope to understand — and potentially disrupt — malignant cycles of uncontrolled cellular growth, replication, and mediated cell death [5]. Effective cancer drug development involves determining how proteins and complexes that are affected by a drug in turn affect important cellular pathways. In this domain, the “downstream” consequences of a particular drug effect are especially important [6]. Stem-cell researchers can also use pathways as an active part of their research, where the goal is generally to precipitate a desired cellular differentiation into specific cell types [7]. In these contexts, understanding the complex relationships that are encoded in pathway data is paramount.

In the last two decades, as the availability of large stores of data to researchers has increased, analyses that involve hundreds or thousands of genes and gene products have become common. When analyzing such large and complex data, visual representations can be essential, and in many cases static, non-interactive, representations will fail to adequately convey the dynamic nature of a pathway. The complexity and amount of information that needs to be incorporated in a given diagram can also make static representations cluttered and difficult to interpret. Thus, modern applications in these domains employ a wide variety of interactive visualization techniques to allow a user to effectively explore and analyze pathway data.

Developing and designing effective visual analytics applications requires a detailed understanding of the visual analysis tasks that will be performed by a user, and the “user” in this case is a biological researcher in the midst of some analysis relevant to their domain. User tasks can thus be designed and understood best through an in-depth understanding of the nature of information needed by the researcher in the course of their analyses. Some of these tasks may not be known a priori and may be exploratory in nature, where an ideal visualization of pathway data could reveal important new insights to a researcher. A comprehensive understanding of tasks performed by domain researchers in a typical analysis is essential to the design and implementation of an effective visual analytics application [8].

In this work, we present a description and analysis of tasks related to the analysis of biological pathway data. Tasks were derived from interviews with several domain experts in biology. After an introduction to the structure and content of pathway data, we describe the task taxonomy that was constructed from these interviews. We also review visual representations of pathway data in the context of our taxonomy, along with a brief discussion of existing tools which implement those visual representations. Finally, avenues of future research are considered, along with a brief summary of lessons learned from domain experts.

### Biological pathway visualization

Pathway models are an important concept in biological research [5–7]. Visualization techniques and applications are essential tools for researchers who work with complex data, and biological pathways are an active area of visualization research.

A number of surveys exist that describe the large number of existing tools for biological network visualization [9–11]. In this paper we highlight some of the more prominent existing tools and techniques that provide support for the tasks described in our taxonomy. However, this paper is not intended to be a complete survey of biological visualization techniques and applications. Here we look at tools that exemplify typical visualization strategies, including: *ChiBe* [12], *Entourage* [13], *Reactome Pathway Browser* [14], *VisAnt* [15], *MetaViz* [16], and *VitaPad* [17].

Node-link diagrams are the nearly-universal choice of visual representation used in existing applications (exceptions to this rule include *BioFabric* [18]). *Cytoscape* [19] is a popular graph visualization application which was originally designed for biological data, and offers many sophisticated plug-ins that have been developed by the research community, including *Cerebral* [20] and *RenoDoI* [21]. However, node-link representations are one of several ways to visualize graph data, and there are alternative visualization techniques which can be applied to pathway data [22, 23]. For instance, research has shown that matrix visualization techniques outperform node-link diagrams for higher level group based tasks [24, 25]. While matrix techniques are not as effective for certain tasks (such as path-tracing), linked views and hybrid techniques exist, such as *NodeTrix* [26], which combine node-link and matrix representations.

#### Pathway data formats

Pathway data can be stored in a variety of file formats which capture the underlying structure of pathway data. In particular, *BioPAX* [2], *KEGG* [27] and *SBML* [28] are the most popular file standards for storing the complex graph data structures inherent in pathway data.

All three of these popular formats are XML-based and represent data as an ontology. *BioPAX*, in particular, was designed to be a general format for biological pathway data across a variety of domain contexts [2]. *Systems Biology Graph Notation* (SBGN) [29] is a visual standard often used to visualize *BioPAX* and *SBML* file formats. Features particular to *SBGN* include the definition of multiple edge and node types, as well as allowing edges to connect to more than two nodes, resulting in a hypergraph. Other formats are used for the visualization of biological pathways that are not specific to the field of biology. For instance, the *SIF Simple Interaction Format* is used by *Cytoscape* [19] to represent undirected interactions between participants.

### Task taxonomies

The field of visualization has produced a number of *task taxonomies*, which are written in an effort to understand how the various tasks performed by an analyst and user are related to (and enabled by) different visualization tools and techniques, and, conversely, how visualization tools might inform analytic tasks. These taxonomies help to clarify the utility of existing techniques while also providing a low-level template for the design and evaluation of new techniques. Wehrend and Lewis [30] provide one of the earliest visualization task taxonomies, with the goal of “accelerating progress in scientific visualization” by allowing researchers to easily find the right visualization technique for a given problem. Shneiderman [31] defines a “task by data type taxonomy” for information visualization in order to “to sort out the prototypes and guide researchers to new opportunities.” Brehmer and Munzner [8] extend these abstractions by linking high-level and low-level tasks into a multi-level typology, which greatly extends the usefulness of a visualization taxonomy, allowing it to be applied to a wide variety of visualization domains.

These seminal taxonomies were, like many later taxonomies, independent of a specific visualization application domain, and their purpose was to provide a low level description and categorization of the analysis tasks enabled by *any* visualization of data. These early taxonomies were written as very general classifications of low level analytic tasks related to any data visualization. In more recent publications, and as visualization research has progressed, task taxonomies have increasingly focused on more constrained subsets of tasks related to particular types of data structures and analytic domains.

More recent taxonomies tend to focus on more narrow categories and domains relevant to visualization. For instance, Valiati et al. [32] provide a taxonomy focused specifically on multidimensional visualizations. They build on earlier work by Wehrend and Lewis [30], but focus on tasks uniquely related to multidimensional visualizations (such as parallel coordinates). Like previous authors, their goal is to guide the choices of visualization and interaction techniques, and also to help support usability testing. Lee et al. [33] define a taxonomy of graph visualization tasks that are frequently encountered when analyzing graph data. The stated goal of this work was to improve the evaluation of graph visualization systems by creating a set of common benchmark tasks (which could be used in conjunction with benchmark data sets). Their taxonomy covers tasks for the analysis of graphs in general, and was inspired by example tasks from several different domains that make regular use of graph data. The authors build on Amar and Stasko’s [34] list of visual analytic tasks by composing existing low-level tasks into higher-level task compositions, while also proposing additional tasks that are not captured by low-level tasks presented in existing taxonomies.

Several recent taxonomies focus on aspects of graph visualization that extend the work of Lee et al. [33]. For instance, Ahn et al. [35] provide a task taxonomy for the analysis of networks that evolve over time, also known as dynamic graphs. The complex nature of dynamic graph data yields a similarly complex set of analysis tasks, and many of these tasks were not covered by the general graph taxonomy of Lee et al. — thus, new tasks needed to be specified. Pretorius et al. [36] focus on multivariate graph visualization (where graph elements contain multiple attributes). Their work builds on the work of both Lee et al. and of Valiati et al. [32], as multivariate networks can be considered a multidimensional dataset. The authors of *enRoute* [37] include a brief discussion of requirements related to their application. Their requirements are somewhat similar to a subset of our tasks, but were created in order to address the technical challenges involved in building *enRoute*, which is specifically used for the analysis of experimental data.

Aside from explicit task taxonomies, several contemporary surveys and state-of-the-art reports are worth mentioning. Hadlak et al. [38] provide a survey of faceted graph visualization techniques, categorizing visualizations based on how the data is faceted, e.g. by attribute, time, or space, and Vehlow et al. [39] survey a variety of techniques for representing groups in graph structures.

While these recently-published task taxonomies have focused on particular data structures (or datasets with particular characteristics), to our knowledge the present work is the first taxonomy of tasks written in the context of the domain of biological pathway analysis.

The nearest existing work is that of Saraiya et al. [4], which builds off of previous work by Saraiya et al. [40], and which involves feedback from domain experts, who evaluate existing pathway evaluation systems. While Saraiya et al.’s [4] objectives are similar to ours, their work differs in several important ways. They approach the taxonomy from the systems perspective, where existing pathway analysis applications are evaluated by domain experts. Here, we focus first on the needs of the domain experts in the context of their real-world research, independently of any specific application or existing visualization system. Finally, the tools evaluated by Saraiya et al. [4] are now over a decade old, and the landscape of visualization tools and techniques has evolved considerably, which justifies a renewed evaluation of pathway analysis tasks.

In this work we focus more on the tasks themselves and look not only at existing biological visualization applications, but at general visualizations and techniques which may be useful in supporting the tasks. Biological pathway visualization is a complex application domain that poses many specific analytic challenges that are not encountered in pre-existing task taxonomies. The data structures underlying biological pathways are dynamic multivariate hyper-graphs, and are more complex than any of those described in previously-published taxonomies. The tasks to be completed by biologists are also highly complex, involving many different entity and relationship types, and are not fully covered by the existing taxonomies.

### Methods

Interviews were conducted with seven domain experts in biology, each of whom works with pathway data in some form. A summary of the interviews is described in Table 1. The domain experts are engaged in a wide variety of research within the general domain of biology and bioinformatics research, but all of which have some relationship to pathway data. Those interviewed included one tenured professor, three assistant professors, one researcher at a cancer research institution, one postdoctoral research associate, and one masters student in bioinformatics. This variety allowed for a rich examination of tasks related to biological datasets.

**Table 1:** Researchers interviewed

Title: Distinguished Professor
Domain: Biochemistry and Molecular Genetics
Studies: Mechanisms of cell survival, cell cycle control, metabolism, and genesis of cancer
Title: Assistant Professor
Domain: Biochemistry and Molecular Genetics
Studies: Proteomics, epigenetic maintenance of adult heart function
Title: Assistant Professor
Domain: Computational and Systems Biology
Studies: Cancer cell death
Title: Assistant Professor
Domain: Bioinformatics and Systems Biology
Studies: Evolution, genetic network topology, population genomics
Title: Postdoctoral Research Associate
Domain: Biochemistry and Molecular Genetics
Studies: High-throughput gene expression analysis
Title: Researcher
Domain: Molecular Oncology
Studies: Cancer research
Title: Master’s Student
Domain: Bioinformatics

The interviews were free-form discussions aimed at understanding the research process of each domain expert, the tasks performed by the researcher in the course of a typical analysis, and, importantly, the structure and content of the data used in their published research. They were intentionally open-ended, and were designed to capture a variety of tasks that are seen as important to domain experts. Researchers were prompted for any existing tools used for analysis, as well as for the types of behaviors that they think they would find useful in a pathway analysis framework. Each researcher also presented their views on the utility of pathway data and of pathway diagrams in general.

We have developed a taxonomy of domain-specific visualization tasks based on these interviews. For each task category, we describe examples of how each task is addressed by current biological visualization applications and techniques.

## Results

Biological pathways are represented as weighted, directed, labeled graphs which can include hyper-edges and compound nodes. While existing task taxonomies describe tasks related to the visual analysis of graphs in general [35, 36], the analysis of pathways in the context of biology reveals several important graph-analytic tasks that other works have not described in detail. This taxonomy refines and extends the existing set of tasks associated with the visual analysis of network data in general.

Our taxonomy divides tasks into three broad categories: Attribute, Relation, and Modification tasks. The attribute category includes the identification of attributes (A1), comparison of attributes (A2), and the identification of provenance and uncertainty (A3 and A4). The relationship category includes the identification of relationship attributes (R1), directed relationships (R2), and grouped relationships (R3), as well as the identification of causality, cascading effects, and feedback loops (R4 and R5). The modification category includes tasks related to updating and curating data, including collaborative annotation (M1) and curation (M2). A summary of the taxonomy can be seen in Table 2.

**Table 2:** A summary of the biological pathway visualization task taxonomy

Category	Example task
Attribute tasks	
(A1) Multivariate	Find all up-regulated genes in a biological pathway. Integrate results of a laboratory experiment into existing protein-protein interaction networks.
(A2) Comparison	Compare a biological pathway to a pathway with the same functionality in a reference species.
(A3) Provenance	Determine which studies provides the evidence for a link between two genes.
(A4) Uncertainty	Understand which pathway components have the strongest empirical evidence relationships.
Relationship tasks	
(R1) Attributes	Find all translocations of entities in a given biological pathway.
(R2) Direction	Find the products or output of a biochemical reaction.
(R3) Grouping	Expand a module entity to include all child-entities in the visualization.
(R4) Causality	Find all genes downstream of the currently selected entity, which may be affected by a change in regulation.
(R5) Feedback	Identify potential feedback loops in gene regulation.
Modification tasks	
(M1) Annotate	Update out-of date-information in a pathway data set, or create a personalized pathway relevant to a specialized research topic.
(M2) Curate	Identify errors and update historical data.

### Attribute tasks

The low-level identification of nodes, edges, and their attributes is an essential component of the visual analysis of any graph representation. In the context of biology, the attributes of a node or edge can themselves be complex objects. Here, we highlight three forms of attribute data that are particularly relevant to biological contexts: multivariate data from experimental results, provenance data, and measures of uncertainty. We also discuss the need for the integration of external data sources.

#### (A1) Identify multivariate attributes

**Description**

The entities within a biological pathway can contain many attributes that reflect the state of that entity in a given context, such as an experimental condition. In interviews, researchers stressed the importance of being able to visualize potentially complex experimental data while viewing a pathway. For example, each entity in a pathway can be associated with gene expression levels across several different experimental conditions, and each of these conditions can include an additional temporal dimension [20], meaning that each node (in this example) would be associated with at least three additional dimensions (experimental condition, expression level, and time).

This multivariate data can also apply to relationships between entities, such as when one gene is up-regulated or down-regulated by another gene under different experimental conditions. Indeed, the identification (and comparison) of attributes is closely coupled with the identification (and comparison) of overall topological structure [37].

An additional concern with biological attribute data is the biological *context* of an entity (e.g., a tissue, organ, or species), especially when datasets can contain similar entity types that were measured across a variety of different contexts.

**Existing approaches and techniques**

Most applications provide access to the attributes through simple interactions (e.g., mouseover and click). In many cases the attribute information is simply read from an input file, however more recent tools such as *SBGNViz* [41] and *ChiBE* [12] query online databases to provide a range of important attribute information.

Multivariate network visualization is a highly active field of visualization, in which the life sciences in general are a frequent application domain, and many more recent biological network visualizations include attribute information. *ChiBE* [12] provides the ability to load biological entity regulation data mappings from an external source and apply them to a pathway visualization. The SIF data format, which is defined as part of the *Cytoscape* application [19], supports these additional data mappings by design. The *RenoDoI* application [21], a plug-in for *Cytoscape* for visualizing knowledge networks of biological data, uses “degree of interest” functions to highlight nodes based on attribute values. Such functionality could easily be extended to biological pathway visualizations. The general purpose visualization system, *Candid* [42] also uses attribute information as part of a hypergraph query system which allows users to perform complex queries on entities of different types. Node and edge attributes are also used for graph querying and filtering as can be seen in facet-based visualizations, an approach that allows for graphs to be filtered by subsets of attributes. The *Cerebral* application [20] uses attribute information as an aid to layout, where the graph layout space is divided into layers and nodes are positioned in the layers based on sub-cellular localization metadata.

Van den Elzen and van Wijk’s [43] system for multivariate graph visualization provides much interactive functionality to aid with the analysis of multivariate data in a graph structure. It aggregates data and provides summary visualization such as histograms and scatter plots that are integrated into graphs visualizing aggregations of a larger network data set. The authors also use widgets that show a visual hint of the underlying data. These widgets, often referred to as “scented widgets” [44], aid interaction with the graph by attributes, and emphasize the importance of the multivariate data in the application.

#### (A2) Compare attributes

**Description**

Related to the issue of multivariate attributes is the need to compare related pathways or sets of entities, or to compare a given pathway across a number of states. For instance, one of the researchers we interviewed described their use of microarray measurements, which are often used to measure gene expression levels for a control group and an experimental group over several time steps. The goal of this research is to discover significant empirical differences between groups and across time, and the visual comparison of these groups is an essential part of an analysis.

In addition, analysts often want to reason about the same entity (e.g., the same protein, gene, or drug) across multiple pathways. In other words, the role or behavior of a biological entity in multiple different contexts is often important.

Visualizations of comparative differences can also be closely coupled with common bioinformatic algorithms. For example, the algorithmic task of discovering subsets of a pathway dataset that are differentially regulated in a given biological context is an important computational problem, and is inherently a comparison task.

The topic of contextualization includes a very important component of modern biology, which is the incorporation of multiple external datasets. Biological pathway data is inherently large, complex, and subject to ongoing contributions from contemporary research. Thus, for biological pathway visualization in particular, integration of attribute data from external data sources is essential.

**Existing approaches and techniques**

In their 2011 survey, Gleicher et al. [45] describe three primary types classifications of comparative visualization. These are juxtaposition, superposition, and explicit encoding of differences, and these classifications can also be combined. A juxtaposition refers to visualizations that are displayed side-by-side in order to facilitate comparison. This is functionality is available by default in *Cytoscape* [19] (and hence all of the associated plug-ins) via simply arranging the windows which display the networks. *Cerebral* [20] uses a juxtaposition approach to display changes in attributes associated with the graph.

Superposition is a technique that involves the display of multiple datasets as part of the same visualization. Within *Cytoscape* there are several ways to map graphical attributes to data, to allow for data from different data sets to be visualized differently. The *RenoDoI* plugin [21] uses superposition as a comparison technique, allowing multiple networks to be visualized in a single image. Bounding isocontours are used to distinguish graphs differences, and to clearly indicate where the graphs overlap. Graph layout is an important aspect of both juxtaposition and superposition based comparative visualizations. Juxtaposition involves comparing two or more graphs using similar layouts in order to aid comparison. For superposition, the matter is not so simple, as the addition of a new graph may destroy the existing layout. The *RenoDoI* application initially lays out the largest data set, then adds the additional data sets, adjusting the previous layout without resetting it. Nodes which are included in both data sets only appear once.

Explicit encoding of difference means that differences between the two datasets are explicitly highlighted, and this approach is often provided in addition to the previous two. For example, an edge which appears in one data set but not the other may be highlighted by color. One specific case where implicit encoding is not mixed with other approaches is seen when a graph is dynamic and the changes are between time slices. This can be seen in Rugfiange and McGuffins’s *DiffAni* application [46] for visualizing dynamic graphs.

#### (A3, A4) Identify provenance and uncertainty

**Description**

Especially important to researchers in the field of bioinformatics is the concept of *data provenance*, which refers to the history of original sources tied to a particular entity. The provenance can refer to the type of source, such as a peer reviewed publication, experimental results, or a textual analysis. Much of the data in the field of bioinformatics is gathered and integrated from a wide range of publications, data stores, and other products of research. Information related to a single entity can be based on potentially dozens of different publications that have been produced across a wide range of time. For example, each relationship within a *BioPAX* file is usually associated with a publication that provides evidence for its existence. The task of visually identifying provenance is complicated in two ways. First, each piece of research related to a given biological entity may corroborate, extend, or contradict earlier publications. Second, the biological context under which a particular entity is studied often varies. The individual studies related to a given gene or gene product might have incorporated cells taken from a variety of tissues, organs, and species. Thus, the provenance information related to a given biological entity can be seen as a temporal network of provenance data, with each publication being tied to earlier works in a variety of ways.

Related to the task of identifying data provenance is the task of being able to understand degrees of *uncertainty* with regards to the underlying data related to entities and their relationships. Biology is different from many other application domains of visualization, as the data is often ambiguous or not certain [47]. The uncertainty can be related to the values of specific attributes or to the existence of a relationship. In their state-of-the-art report on the visualization of group structures in graphs, Vehlow et al. [39] discuss uncertainty as one of several ongoing research challenges. The importance of understanding uncertainty was emphasized by several of the researchers we interviewed. Uncertainty may relate directly to the provenance history discussed above — biological entities that are related to more recent research may have a limited set of one or two publications which corroborate their functionality, while other genes and gene products may have a rich history of robust empirical evidence from dozens or hundreds of publications. An even more fine-grained approach to uncertainty visualization could incorporate the uncertainty or error tied to individual empirical findings and experimental results. The empirical support behind any individual entity or relationship within a pathway can vary widely, and the question of how these varying levels of confidence can be incorporated into a pathway visualization has been rarely addressed.

**Existing approaches and techniques**

While *SBGNViz* [41] and *ChiBE* [12] and other applications allow connectivity to external sources, such as *UniProt* or *PubMed*, there are few biological visualization tools that visualize provenance information directly.

Most online biology databases do provide this information but do not integrate it into the data visualization itself. For example, *Reactome* [14] displays a list of publications which are related to the selected item as a simple list in a separate window adjacent to the visualization.

*STRING* [48], a protein interaction database, provides provenance information and incorporates it into its associated visualization. The provenance is described with respect to its source (e.g., experimental results or a curated database) and is encoded by color within the database’s visualization component. *BranchingSets* [49] uses multi-colored links and nodes to indicate the provenance of specific proteins and biochemical relationships between proteins, making it easier for a user to see which contexts are relevant for particular elements. *TimeArcs* [50] is a visualization technique that highlights PubMed articles related to particular subnetworks of proteins within a specified time range. At a glance, a user can see whether or not a particular protein or set of proteins is described within the literature of biological pathways. Moreover, he or she can see if the relationships between each of these proteins is confirmed or contradicted by successive publications, indicating, for example, further details about known pathways, or that in different contexts (e.g., tissues, organs, or species) pathways exhibit different functionality.

Some databases also provide quality scores with their results. This quality score can be seen as a form of uncertainty as it relates to the amount of information available concerning a relationship or entity. The higher the score, the more evidence there is for an interaction.

Visualizing uncertainty and ambiguity is still a challenge in visualization in general. There are many different types of uncertainty [51]. In biological visualization uncertainty may be caused by measurement errors, missing data, algorithms providing multiple solutions (only one of which is used in the resulting data set) and ambiguous mapping between elements in different domains [47].

One characteristic of uncertainty within an analysis is that it can build over time. As a researcher filters and adds external data to a biological pathway visualization the amount of uncertainty present in the visualization as a whole will change. An approach similar to the uncertainty flows of Wu et al. [52] could be used to help researchers comprehend the impact of their decisions on overall uncertainty levels when creating a biological pathway visualization.

Visualizing uncertainty within a graph visualization is an ongoing challenge in the domain of visualization, with few practical examples available. Wang et al. [53] use a variant of a heat map visualization to show where visual ambiguity occurs in a graph visualization. While their approach visualizes potential ambiguity in visual interpretation rather than within the underlying data set, a similar approach could be taken to visualize uncertainty in biological networks.

### Relationship tasks

Within bioinformatics, understanding relationships within a biological pathway graph is one the most essential tasks that a systems biologist will perform. All of the researchers we interviewed stressed the importance of understanding how pathway entities within a biological network are connected. Here, we discuss some of the complex types of relationships found within biological datasets. We emphasize that the challenge of visualization is not only that these different categories of relationship exist, but that they exist as combinations and compositions of each other.

#### (R1) Identify relationship attributes

**Description**

One of the most obvious challenges for biological network visualization is the fact that the types of relationship between entities are numerous, and even hierarchical. For instance, an interaction between two entities could take many forms, including: the binding of proteins and molecules into complexes, the translocation of an entity from one cellular location to another, a change in gene expression activity, or the modification of existing compounds, to name a few. Each of these events can be further specified. For example, a modification can take many forms, such as *ubiquitination* or *phosphorylation*, and the *site* at which these modifications occur can also be specified. Changes in gene expression are directional — one compound can either *increase* or *decrease* the activity of another. A translocation event will typically specify *from* and *to* locations. Thus, not only are there many different types of relationship (and generally more than can be effectively encoded using color alone), but each relationship type has its own set of potential specifications, some of which can be quite detailed.

**Existing approaches and techniques**

The visual encoding of these complex and multivariate relationships is one of the more prominent challenges in the design of visual analytic platforms for biological pathway analysis.

Pretorius and van Wijk’s [54] system for visual inspection of multivariate graphs places the relationship type (referred to as edge labels) at the core of their system. They do not use traditional graph layout techniques, and their resulting visualization resembles the parallel coordinates style of multivariate data visualization. The edges are grouped by label in the center of the display, nodes are duplicated on either side, with the attributes reflected by an icicle plot. This approach can handle a large number of edge types, and cases where a node is involved in multiple relationships of different types.

Ghani et al. [55] developed a techniques called *Parallel Node-Link Bands* (PNLBs) for exploring graphs with multiple edge types. In their examples, edge types are inferred based on their endpoint node types. Nodes are listed in vertical columns with the edges connecting only between neighboring columns. This technique is similar to Pretorious and van Wijk’s approach except that there are multiple columns of nodes and there is only ever one type of edge between two columns. It is an effective visualization, but is generally limited to smaller data sets and those in which the relationship types are multiple bimodal relationships (as there are no edges drawn between non-adjacent columns).

#### (R2) Identify directed relationships

**Description**

While some analyses and datasets involve undirected relationships between genes or gene products, the majority of studies of metabolic networks and other inter-cellular processes rely on directed relationships. Several researchers that we interviewed stressed the importance of understanding directed relationships between entities. Depending on the type of relationship in question, edges may be *bi-directional*, which is distinct from an *undirected* edge. A visual coding that indicates direction must also be able to account for cases in which there are two directional edges between the same two nodes.

**Existing approaches and techniques**

Many visualization applications use the more traditional approach of arrowheads to indicate edge directions, however work by Holten and van Wijk [56] shows that tapered edges perform more effectively in conveying edge direction. The graphs used in Holten and van Wijk’s are simple directed graphs. Biological pathways are usually modeled as hyper-graphs, with many different types of edges and hyperedges. Visual encodings such as *SBGN* and *KEGG* contain many different visual representations for edges, so applying the tapered edge visualization style to complex biological pathways is not trivial and would require an empirical evaluation. However, the results of Holten and van Wijk’s work suggest that investigating such an approach may be worthwhile.

#### (R3) Identify grouping / hierarchical relationships

**Description**

Pathway data is inherently hierarchical, and there are many ways in which nodes can be grouped into collections of elements that are related in an explicit biochemical sense (e.g., complex proteins) or in a more implicit informational sense (e.g., the biochemical reactions related to a higher-order biological process). Grouping relationships describe relationships of containment, and these relationships can be abstract or based on real biochemical interactions within a cell. For example, a pathway (itself an abstraction) can be nested within other pathways. These nested pathways generally encapsulate some commonly-understood hierarchy of biological processes that take place within a cell, such as cellular replication. Other representations include the more general notion of a module of connected components, such as gene products. Grouping relationships can also represent physical interactions between biochemical participants. A common of example of this is in biomolecular complexes, which are themselves composed of other complexes or biomolecules.

It is important to note that hierarchy and “structure” often co-exist with other types of relationships. In most cases, pathway data includes relationships of hierarchy (i.e., when one vertex is contained within another) *in parallel* with other, non-hierarchical relationships, such as the relationship between one gene product that activates or inhibits another. Also, note that while non-hierarchical relationships can take a variety of forms, the only form of hierarchical relationship is one of *containment*, from parent to child, and is undirected.

Grouping relationships also include the concept of *compound* nodes. A vertex that contains other entities can be represented as a *compound node*, which is equivalent to a parent vertex or in some contexts a “module.” It is important to note that a one-to-one relationship between an entity and a parent is *not* the same as a one-to-many relationship between an entity and all of that parent’s children. For instance, the *BioPax* format allows for the abstract *NextStep* relationship, which defines, as the name suggests, an arbitrary notion of the next step of some biological process. A biochemical reaction could be connected, via a single *NextStep* relationship, to an entire pathway, which could potentially contain thousands of nodes. This relationship is clearly not the same as a biochemical reaction being connected to every entity within a pathway. This example also demonstrates the distinction between a compound relationship and a hierarchical relationship (which are two types of grouping relationships). A connection from a node to a compound node does not imply a relationship of ownership or containment.

**Existing approaches and techniques**

There are a variety of visualization techniques for the display of “grouped” nodes and hierarchical data. Numerous tree based graph layouts position nodes to emphasize the hierarchical nature of data, however these are often not suitable for biological pathway layout as the constrains on position in a layout affect the readability of the lowest level of information. The *RenoDoI* [21] application allows for multiple data sources to be included in a single diagram. This containment relationship may include data from different pathways. In this system, the node for each data source forms a set, which may or may not overlap with other sets. This is visualized by drawing a bounded contour around the nodes in the set, where different border colors indicate different sets. This type of encoding of set membership is the *Bubble Sets* [57] approach, which was shown to be the most effective way of displaying group information on a node-link diagram by Jianu et al. [58].

The *BranchingSets* technique [49, 59] facilitates the exploration of hierarchical information in biological pathways, which is presented directly within the nodes in network. At a glance, a user can see an overview of the nested structure of a protein complex, and user interaction brings up a more elaborate tree view that provides further details about a selected complex, highlighting the hierarchical patterns within a set of pathways.

#### (R4, R5) Identify causality and cascading effects

**Description**

A category of tasks inherent to a variety of work in bioinformatics is the identification of *causal relationships* that exist between biomolecular entities, and *causal networks* are of particular importance to the analysis of large-scale gene expression data.

When discussing directed paths between entities, one entity is said to be *upstream* or *downstream* of another. For example, one gene product can increase the activity of other gene products that are *downstream* of it. Understanding these upstream and downstream relationships is particularly important to domains such as cancer drug research, where a drug may affect a small subset of genes or gene products, which in turn will affect various downstream processes. In most cases, a directed relationship is meant to represent a biochemical reaction, where one entity is consumed as a reactant and another is produced as a product. Thus, an upstream entity may be connected to a downstream entity through a chain of several directed links, and a researcher may be interested in understanding the path of reactions (or other relationships) that connects two entities. However, most cellular processes are inherently complex, and involve many competing sets of directed interactions. Any given gene is often *mediated* by many different reactants, some of which increase activity, and others which decrease activity. For instance, a causal network helps to reveal the likely regulators of a set of genes that are observed to be up-regulated or down-regulated in a particular setting [60, 61].

Thus, determining the set of entities that are “responsible” for the increase or decrease in the expression of a particular gene is a challenging task that involves a complex array of directed relationships between many upstream entities. We characterize this problem as one of identifying *cascading effects*, where many upstream entities have directed relationships with many mediating entities, which in turn affect the output of many downstream entities.

In tandem with the problem of identifying cascading effects is the problem of reasoning about feedback [23]. Feedback loops are common within metabolic activation networks, and they play a key role in processes related to uncontrolled cellular growth in cancerous cells [5].

Causality and cascading effects depend on the both the structure of the graph, which determines the global propagation of change, and the attributes associated with individual graph entities, e.g., a change in a particular gene expression level from being up-regulated to down-regulated. In this case, the structure of the graph does not change, only entity attributes (which Ahn et al. [35] refer to as the domain properties). Archambault et al., in their definition of temporal multivariate networks [62], describe these changes in attributes as the behavior of the graph. They also note that high attribute dimensionality is still an open problem for temporal multivariate networks. Causality can be closely coupled with network topology, and the these two concerns will often need to be analyzed jointly, as discussed by the authors of *enRoute* [37].

**Existing approaches and techniques**

Showing the full range of behaviors (attribute value changes) in a traditional biological pathway network visualization can be difficult as there are relatively few visual encodings which can indicate attribute values (e.g., color, shape, texture, etc.). The approach of Pretorius and van Wijk [54] allows for a large number of attributes to be displayed, but differs hugely from traditional biological pathway visualization approaches in that it shows little overall structure. However this approach, or one influenced by it, might be beneficial if used in conjunction with another view of the pathway which clearly shows the structure which propagates the changes.

With respect to cascading changes of attributes, Archambault and Purchase [63] have performed a empirical evaluation of several different techniques. They found that the use of *small multiples* seems to be the best approach to convey the dynamic attribute changes that cascade through a network. The small multiples approach is a form of comparative juxtaposition where multiple views of the network at different time points are displayed in a matrix. This approach has been used by the *Cerebral* application for showing cascades of data [20]. Archambault and Purchase’s work also shows that layout has an impact on the visualization of attribute cascades. Participants in the experiment performed better when a hierarchical layout was used, however it should be noted that the hierarchical layout was consistent with the direction of the cascade. Additionally, the authors of *enRoute* [37] briefly discuss a case study in which their tool can be used for the visualization of causality in the context of experimental results.

### Data modification

While most of the tasks in this taxonomy are directly related to visual analysis, the size and complexity of biological datasets makes data curation an essential part of modern research platforms.

#### (M1, M2) Annotate and curate

**Description**

Several of the researchers we interviewed mentioned certain tasks related to the curation, maintenance, and understanding of pathway data. For instance, one researcher mentioned the importance of being able to *debug* potentially flawed data. Two others expressed a need to create “personalized” pathways that only include a user-determined subset of entities and relationships. Ideally, visualization tools will seamlessly integrate these curation and maintenance needs.

An important aspect of data modification is the notion of collaboration — where several researchers are allowed, synchronously or asynchronously, to modify and update a dataset. The concept of collaboration is increasingly important as more analytics platforms move to the web, and the topic of effective user-centered design for scientific collaboration will become increasingly relevant in the future.

The topic of contextualization includes a very important component of modern biology, which is the incorporation of multiple external datasets. Biological pathway data is inherently large, complex, and subject to ongoing contributions from contemporary research. Thus, for biological pathway visualization in particular, integration of attribute data from external data sources is essential.

**Existing Approaches and Techniques** Most desktop pathway visualization applications allow for data files to be edited and exported in standardized formats, e.g., *CellDesigner* [64] allows files to be modified and curated and exported in the *SBML* standard. Saving a personalized version of a pathway is basic functionality, but curating a large data set may take the input of many experts. Collaborative online visualizations such as *Polychrome* [65] allow a synchronized viewing of a web-based visualization across multiple users (and across multiple devices). Collaborative web-based visualizations also offer an opportunity for researchers to share their personally curated pathways and data sets for generally dissemination or for support in debugging possibly flawed pathways. The ability to disseminate biological pathway visualizations easily amongst multiple curators would allow for a more thorough validation of proposed pathways.

The approach of using web tools to disseminates knowledge is already very evident in many modern online biological data resources. *Reactome* [14] and *STRING* [48] are two online resources which feature highly interactive web based visualization interfaces. The fact that the data in the *Reactome* database is curated and peer reviewed is considered an important feature of the system. Most online publicly available pathway databases do allow users to provide feedback on potential errors, updated research, or areas for improvement.

## Discussion

In the process of creating a domain-specific task taxonomy that is based on interviews with domain experts, we have revealed several tasks that are important to domain researchers but which merit further attention and more focused scrutiny from the visualization community. While tools exist which support these tasks, existing approaches tend to be ad hoc and may lack features that would be revealed by more user-centered design methodologies. However, we do not mean to suggest that future research should be constrained to these tasks alone, but that they should be given more focused attention in the field of pathway visualization research. Below we outline some directions for future research.

**Visualizing causality and cascading effects**

Previous taxonomies only touch on the notion of network-level causality and cascading effects in passing. Lee et al. [33] describe a set of attribute-based tasks in their taxonomy, but they do not consider causal or cascading relationships, only tasks related to static attribute values. Ahn et al. [35] in their task taxonomy for network evolution analysis do describe the notion of attribute stability as part of the shape of changes in their taxonomy, and describe tasks that iterate and enable each other, but there is no classification of causality or cascades at the attribute level. In their overview of dynamic network visualization, Moody et al. [66] do mention cascades, but only in the context of the formation of relationships, not the changing of attributes. Attribute cascades and feedback loops are a very important aspect of biological pathway visualization, and merit further attention. The problem of visualizing dynamism and causality in networks in an open problem in information visualization, and is of particular importance to researchers in bioinformatics. A careful study of techniques for visualizing causality in the context of complex biological datasets would be a valuable contribution to the visualization literature.

**Visualizing uncertainty and provenance**

Many visualization tools generally do not attempt to visualize the uncertainty behind a connection in a pathway, which was particularly important to many of the domain experts we interviewed. Uncertainty may exist as a result of experimental measures (i.e., statistical uncertainty) or as a result of varying provenance. In bioinformatics, provenance is an important and complex layer of metadata that accompanies any dataset. Furthermore, data provenance was mentioned as a particularly important concern by most of the domain researchers we interviewed, but there are relatively few examples of visual analytics tools which explore provenance visualization in a direct and robust way. Visualizing provenance and uncertainty is a challenging task, as even the definition of uncertainty may be difficult to operationalize, and each datum in a given pathway could be associated with a potentially large and complex hierarchy of research. However, data formats such as *BioPAX* do have robust support for citations, allowing published references to be connected to entities and relationships within a pathway. A tool that could effectively encode data related to uncertainty and provenance into a visualization would be very valuable to systems researchers who work with the results of hundreds or thousands of publications and experimental datasets.

### Limitations and continued research

As mentioned earlier, our interviews with domain experts were intentionally open-ended and a structured interview process (such as grounded theory) was not used. This format was used in order to encourage a diverse assortment of feedback from researchers who engage in a wide variety of research within biology and bioinformatics. As a result of this free-form structure, our tasks identified in this taxonomy are by no means meant to be an exhaustive list of all possible visualization tasks — it is certainly possible for this taxonomy to be refined and extended, and additional contributions and suggestions are welcome. In addition, a second round of explicitly structured interviews will be a valuable next step in the continued refinement of this taxonomy. Our interviews have created a valuable base of user feedback that has guided the identification of tasks that are of particular importance to domain experts.

## Conclusions

While a wide variety of pathway visualization tools exist, there is still plenty of room for innovative platform development. Many tools tend to greatly overlap each other with respect to the analytical tasks available, and attempts to directly address the most challenging aspects of pathway data analysis are few and far between. Having a detailed understanding of the tasks performed by researchers who work with pathway data is essential to the development of effective visual analytics platforms for pathway analysis.

While relevant network visualization taxonomies exist, they largely describe tasks related to network data visualization in *any* context. While our goal in creating this taxonomy was to identify visualization tasks relevant to researchers who work with biological pathway data, our taxonomy also acts as a template for domain-specific task taxonomies in the more general context of information visualization research. Existing visualization contexts are often deliberately generalized, and are meant to address challenges and provide guidelines for the visualization of certain categories of *data*, rather than certain categories of *research*. While such generalization is obviously useful, we have shown that the opposite approach can be useful as well. By focusing on specific research domains, visualization researchers can capture the needs of domain researchers, allowing them to build more user-centric task taxonomies. These domain-specific taxonomies have the potential to reveal real-world tasks that may not have been adequately captured by more generalized taxonomies.

Here, we have built a task taxonomy by starting with low level research tasks identified by domain experts as important to their research. As the field of biological pathway visualization continues to grow, we hope to emphasize the needs of real-world domain experts, and to guide researchers towards avenues of research that could lead to valuable contributions. Through interviews with domain experts, we have identified tasks important to researchers who work with biological pathway data, and this taxonomy acts as a reference point for the large and growing field of biological pathway visualization. The continued refinement and understanding of tasks related to pathway data in bioinformatics research will create an important foundation for the ongoing development of pathway visualization.
